# Ferrous Sulfate and Folic Acid Supplementation in Pregnant People in Primary Health Care: a Cross-Sectional Study, Criciúma, 2022

**DOI:** 10.1590/S2237-96222026v35e20240377.en

**Published:** 2025-11-28

**Authors:** Jhonatan Campos-Rosa, Ricardo Augusto Cardoso Puchivailo, Antônio Augusto Schäfer, Fernanda de Oliveira Meller, Vanessa Iribarrem Avena Miranda

**Affiliations:** 1 Universidade do Extremo Sul Catarinense, Curso de medicina, Criciúma, SC, Brazil; 2 Universidade do Extremo Sul Catarinense, Programa de Pós-graduação em Saúde Coletiva, Criciúma, SC, Brazil

**Keywords:** Pregnancy, Folic Acid, Primary Health Care, Ferrous Sulfate, Cross-Sectional Studies, Embarazo, Ácido Fólico, Atención Primaria de Salud, Sulfato Ferroso, Estudios Transversales

## Abstract

**Objective:**

To evaluate the prevalence and factors associated with supplementation of ferrous sulfate and folic acid in pregnant people.

**Methods:**

A cross-sectional, representative study with pregnant people treated in Primary Health Care in Criciúma, Santa Catarina, in 2023. Ferrous sulfate and folic acid supplements were evaluated based on the prenatal booklet for pregnant people. The prevalence of supplementation was calculated with their respective 95% confidence intervals (95%CI). Crude and adjusted Poisson regression analyses (hierarchical model) were performed, estimating prevalence ratios (PR) and their respective 95%CI, to verify the associated factors.

**Results:**

A total of 428 pregnant people were interviewed. Of these, 97.4% used ferrous sulfate supplementation during pregnancy, and 93.7% folic acid. The analyses indicated that the use of ferrous sulfate was higher in older pregnant people, with PR 1.06 (95%CI 0.99; 1.13), and who consumed alcohol during pregnancy, with PR 1.04 (95%CI 1.00; 1.08). Regarding the use of folic acid, it was higher among pregnant people who had at least six prenatal visits, with PR 1.10 (95%CI 1.01; 1.20).

**Conclusion:**

The results indicate high iron and folic acid supplementation among pregnant people in Primary Health Care. The identification of associated factors can guide more targeted and effective intervention strategies by Health Management.

## Resumen

**Objetivo:**

Evaluar la prevalencia y los factores asociados a la suplementación con sulfato ferroso y ácido fólico en personas embarazadas.

**Métodos:**

Estudio transversal, representativo, con personas embarazadas atendidas en la Atención Primaria de Salud de Criciúma, Santa Catarina, en el año 2023. Las suplementaciones con sulfato ferroso y ácido fólico se evaluaron a partir de la cartilla prenatal del embarazo. La prevalencia de suplementación se calculó con sus respectivos intervalos de confianza del 95% (IC95%). Se realizaron análisis de regresión de Poisson crudos y ajustados (modelo jerárquico), estimándose razones de prevalencia (RP) y sus respectivos IC95% para verificar los factores asociados.

**Resultados:**

Se entrevistaron 428 personas embarazadas. De ellas, el 97,4% utilizó suplementación con sulfato ferroso durante la gestación y el 93,7% con ácido fólico. Los análisis indicaron que el uso de sulfato ferroso fue mayor entre las personas embarazadas de mayor edad, con RP 1,06 (IC95% 0,99; 1,13), y entre aquellas que consumieron bebidas alcohólicas durante la gestación, con RP 1,04 (IC95% 1,00; 1,08). En cuanto al uso de ácido fólico, este fue superior entre las personas embarazadas que realizaron al menos seis consultas prenatales, con RP 1,10 (IC95% 1,01; 1,20).

**Conclusión:**

Los resultados indican una alta prevalencia de suplementación con hierro y ácido fólico entre las personas embarazadas atendidas en la Atención Primaria de Salud. La identificación de los factores asociados puede orientar estrategias de intervención más específicas y eficaces por parte de la gestión sanitaria.

**Ethical aspects**

This research respected ethical principles, having obtained the following approval data:

Research Ethics Committee: Universidade do Extremo Sul Catarinense

Opinion number: 5,053,755

Approval date: 22/10/2021

Certificate of Submission for Ethical Appraisal: 52547521.0.0000.0119

Informed Consent Form: Obtained from all participants prior to data collection.

## Introduction 

Pregnancy is a period of intense physiological changes in which various nutritional needs are increased to ensure the healthy development of the fetus and maintain the health of the pregnant person (1,2). Among the essential nutrients, iron and folic acid play a fundamental role (2). Iron deficiency can lead to iron deficiency anemia. This multifactorial condition manifests itself when there is a reduction in the amount of hemoglobin or erythrocytes, resulting in a decrease in the blood’s ability to transport oxygen through the body (3). Folic acid deficiency during the prenatal period, in turn, can also impair the maternal-fetal dyad, as it plays a primary role in the formation of the neural tube of the fetus, which develops in the first weeks of pregnancy, and which will become the baby’s brain and spinal cord (4,5).

Ideally, most nutrients should be obtained through a balanced diet. However, iron and folic acid needs during pregnancy can be difficult to meet through diet alone. For this reason and due to the high prevalence of anemia in pregnant people, especially in developing countries, several governmental organizations recommend supplementation of these micronutrients during pregnancy as a strategy to improve nutritional status and prevent possible deficiencies (6,7). 

In Brazil, the National Iron Supplementation Program (*Programa Nacional de Suplementação de Ferro*, PNSF), implemented in 2005, recommends the daily intake of folic acid for at least 30 days before conception and until the 12th week of pregnancy (8). As for iron, the recommendation is its daily administration from the confirmation of pregnancy until the third month after delivery (9,10).

Primary Health Care plays a central role within the Brazilian National Health System (*Sistema Único de Saúde*, SUS), being the main means of access to health care services for the population (11,12). It is the responsibility of the Primary Health Care team to carry out risk stratification during prenatal visits, classifying pregnant people according to their specific characteristics and preventing possible nutritional deficiencies. (13). However, even with a universal coverage program, studies indicate that daily supplementation is below the recommended levels, with 40 mg of iron and 0.4 mg of folic acid being advised (2,8,9). Data from the cohort study, which evaluated 4,200 pregnant people, showed that the use of folic acid is more frequent among White women, with higher income and higher education level. In contrast, the prophylactic use of iron was more common among non-White and poorer mothers with lower education level, suggesting the opposite association for this supplement (14).

Several studies report the use of supplements during pregnancy. However, few have focused on Primary Health Care (14-16). Although there is a universal coverage program in Brazil for the use of folic acid and ferrous sulfate during pregnancy, the literature points out that the use of these compounds may be lower than expected (17). As a strategy to evaluate the program’s performance, the present study aims to investigate the prevalence and factors associated with supplementation of ferrous sulfate and folic acid for pregnant people treated in Primary Health Care.

## Methods 

### Design 

This is a cross-sectional study, from the research entitled “Mental health and living conditions of pregnant people cared for in Primary Health Care,” carried out from April to December 2022. 

### Setting 

Criciúma is located about 200 km south of Florianópolis, capital of Santa Catarina, and covers approximately 234,865 km^2^. Its Human Development Index (HDI) is 0.788. The municipality has a population of 215,186 inhabitants, with an average monthly income of 2.6 minimum wages per person and a Gross Domestic Product (GDP) per capita of BRL 33,811.65 (18).

### Study size

The sample size calculation was performed in the Open Source Epidemiologic Statistics for Public Health (OpenEpi) program with the following parameters: 95% confidence level, 80% statistical power, and 59% outcome prevalence. An additional 10% was added for losses and refusals, and 15% for control of confounding factors, resulting in an estimated sample size of 384 pregnant people.

### Participants 

The target population was composed of pregnant people aged 18 years or more, within the third trimester of pregnancy, and who were receiving their prenatal care in one of the 48 health centers of Criciúma. 

As exclusion criteria, we considered: pregnant people under 18 years of age; pregnant people with cognitive deficit or communication disability that prevented them from answering the questionnaire; pregnant people of another nationality who did not understand Portuguese; pregnant people with high-risk pregnancy and/or cases of abortion.

### Variables 

The outcomes “iron supplementation” and “folic acid supplementation” were obtained from the specific field in the prenatal booklet, which was filled out by the health personnel during routine visits. The socioeconomic variables used were: mother’s age (18–19 years, 20–29 years, and ≥30 years); mother’s “race/skin color” of the pregnant people enrolled in the study (White, Black/Brown); marital status (single, married/stable union, separated); maternal education level (0–4 years, 5–8 years, 9–11 years, and ≥12 years); and monthly income (<BRL 500, BRL 500–1,000, BRL 1,001–2,000, BRL 2,001–4,000, and >BRL 4,000). In addition, pregnancy planning (yes, no) and the number of visits attended during prenatal care (<6 visits and ≥6 visits).

### Data sources and measurement

Data collection was carried out by Community Health Workers and students of the Multiprofessional Residence Program of the Universidade do Extremo Sul Catarinense, who were previously trained and guided in the conduct of data collection and recording. The questionnaires were applied using the Research Electronic Data Capture (RedCap) application through smartphones.

The identification of pregnant people in the third trimester was carried out through the computerized health system used by the municipality, called “CELK Saúde.” In it, monthly reports were generated with the probable date of delivery for the next three months, corresponding to the third trimester of pregnancy. All pregnant people were invited to participate in the study. The interviews were preferably conducted at the health centers, according to the date of the prenatal visit, and, when necessary, were also conducted at the residence of the pregnant person. 

### Bias control 

To minimize possible biases, several methodological strategies were adopted. The selection of participants was carried out systematically, based on monthly reports extracted from the municipal system CELK Saúde, ensuring the inclusion of pregnant people in their third trimester. The collection was conducted by previously trained and standardized professionals, using the RedCap application, which reduced transcription errors. Information on supplementation was extracted from prenatal booklets, standardizing the data source. However, it is acknowledged as a limitation that these records indicate only the prescription, without reflecting actual adherence or regularity of use. 

### Statistical methods

The analyses were performed in Statistical Software for Data Science (STATA) version 17.0, using the survey module for complex samples, incorporating post-stratification weights. Initially, the sample was described concerning the independent variables, and the prevalence of iron and folic acid supplementation outcomes was calculated with the respective 95% confidence intervals (95%CI).

To assess the associations between outcome and explanatory variables, the measure of association used was the prevalence ratio (PR), obtained through Poisson regression models with robust variance, following a hierarchical model. Level 1 included demographic and socioeconomic variables; Level 2 included lifestyle and pregnancy planning variables; and Level 3 included the number of visits. Variables with a p-value<0.2 were maintained in the model to control for confounding factors. A significance level of 0.05 was considered.

## Results 

A total of 428 pregnant people were evaluated (response rate of 85.6%). According to Table 1, the majority belonged to the age group of 20 to 29 years (53.3%), White “race/skin color” (68.1%), and were married or in a stable union (68.2%). Approximately half of them had nine to 11 years of schooling (50.5%). About half had an income of BRL 1,001.00 to BRL 2,000.00 (45.0%). Regarding prenatal care, 79.7% had six or more visits. Most pregnancies were unplanned (62.8%). Regarding lifestyle habits, most pregnant people were non-smokers (95.6%) and did not consume alcohol (97.0%). 

**Table 1:** Sample distribution with 95% confidence interval (95%CI) according to the characteristics of pregnant people attended in Primary Health Care in Criciúma, 2022 (n=428)

Variables	n (%)		(95%CI)
**Age** (years)			
18-19	42 (9.8)	(7.3; 13.0)	
20-29	228 (53.3)	(48.5; 58.0)	
≥30	158 (36.9)	(32.6; 41.6)	
**Race/skin color**			
White	284 (68.1)	(63.5; 72.4)	
Black	48 (11.5)	(8.8; 15.0)	
Brown	85 (20.4)	(16.8; 24.5)	
**Marital status**			
Single	124 (29.0)	(24.9; 33.5)	
Married/Stable Union	292 (68.2)	(63.6; 72.5)	
Separated/Divorced	12 (2.8)	(1.6; 4.9)	
**Schooling** (years)			
0-4	6 (1.4)	(0.6; 3.1)	
9-11	216 (50.5)	(45.7; 55.2)	
≥12	88 (20.5)	(17.0; 24.7)	
**Monthly income** (BRL)			
<500.00	86 (20.8)	(17.2; 25.0)	
500.00-1,000.00	70 (17.0)	(13.6; 20.1)	
1,001.00-2,000.00	186 (45.0)	(40.3; 49.9)	
2,001.00-4,000.00	57 (13.8)	(10.8; 17.5)	
>4,000.00	14 (3.4)	(2.0; 5.6)	
**Number of prenatal visits**			
<6 visits	87 (20.3)	(16.8; 24.4)	
Six or more visits	341 (79.7)	(75.6; 83.2)	
**Planned pregnancy**			
No	269 (62.8)	(58.2; 67.3)	
Yes	159 (37.2)	(32.7; 41.8)	
Parity			
1	129 (48.9)	(42.8; 54.9)	
2	79 (29.9)	(24.6; 35.7)	
3	35 (13.2)	(9.6; 17.9)	
4 or more	21 (8.0)	(5.2; 11.9)	
**Smoking during pregnancy**			
No	409 (95.6)	(93.2; 97.2)	
**Alcohol consumption**			
No	414 (97.0)	(94.8; 98.2)	
Yes	13 (3.0)	(1.8; 5.2)	

During pregnancy, 97.4% of people received ferrous sulfate supplementation, while 93.7% received folic acid. When supplementation was analyzed according to the characteristics of the pregnant people, a direct relationship was observed between age and iron supplement use. As age increased, prevalence also rose, reaching 100% among pregnant people aged 30 years or older (p-value 0.035). Additionally, pregnant people who attended six or more prenatal visits showed higher use of ferrous sulfate, reaching a prevalence of 98.2%. On the other hand, those who had fewer than six visits had a lower prevalence of iron supplementation (94.2%) (p-value 0.036). 

The same trend was observed with folic acid supplementation. Pregnant people who attended six or more prenatal visits had a higher frequency of use, reaching 95.6%, while those with fewer than six visits recorded 86.2% (p-value 0.001). Regarding smoking during pregnancy, pregnant smokers had lower iron supplementation rates (89.5%) compared to non-smokers (97.8%) (p-value 0.025). Regarding folic acid, the same pattern was observed, with pregnant smokers showing a lower frequency of use of 78.9%, compared to 94.4% among non-smokers (p-value 0.007), as shown in Table 2.

**Table 2:** Prevalence of folic acid and iron supplementation according to the characteristics of pregnant people assisted in Primary Health Care in Criciúma, 2022 (n=428)

Variables	Folic acid supplementation		Iron supplementation	
	n (%)	p-value	n (%)	p-value
**Age** (years)		0.461		0.035
18-19	41 (97.6)		40 (95.2)	
20-29	214 (93.9)		219 (96.0)	
≥30	146 (92.4)		158 (100)	
**Race/skin color**		0.300		0.605
White	270 (95.1)		278 (98.9)	
Black	43 (89.6)		46 (95.8)	
Brown	79 (93.0)		82 (96.5)	
**Marital status**		0.320		0.441
Single	117 (94.3)		121 (97.6)	
Married/Stable Union	274 (93.8)		285 (97.6)	
Separated/Divorced	10 (83.3)		11 (91.7)	
**Schooling** (years)		0.469		0.119
0-4	5 (83.3)		5 (83.3)	
5-8	110 (93.2)		114 (96.6)	
9-11	201 (93.1)		211 (97.7)	
≥12	85 (96.6)		87 (98.9)	
**Monthly income** (BRL)		0.614		0.497
<500.00	79 (91.9)		82 (95.35)	
500.00-1,000.00	64 (91.4)		67 (95.7)	
1,001.00-2,000.00	174 (93.6)		183 (98.4)	
2,001.00-4,000.00	55 (96.5)		56 (98.2)	
>4,000.00	14 (100)		14 (100)	
**Number of visits**		0.001		0.036
<6 visits	75 (86.2)		82 (94.2)	
Six or more visits	326 (95.6)		335 (98.2)	
**Planned pregnancy**		0.403		0.956
No	250 (92.9)		262 (97.4)	
Yes	151 (95.0)		155 (97.5)	
Parity		0.063		0.665
1	120 (49.6)		124 (48.6)	
2	74 (30.6)		77 (30.2)	
3	32 (13.2)		33 (12.9)	
4 or more	16 (6.6)		21 (8.2)	
**Smoking during pregnancy**		0.007		0.025
No	386 (94.4)		400 (97.8)	
Yes	15 (78.9)		17 (89.5)	
**Alcohol consumption**		0.837		0.552
No	388 (93.7)		403 (97.3)	
Yes	12 (92.3)		13 (100)	

Table 3 describes the factors associated with supplementation during pregnancy. Regarding folic acid, both crude and adjusted analyses showed an association with an adequate number of prenatal visits. Pregnant people with six or more visits had 10% higher folic acid supplementation compared to those with fewer than six medical visits. Regarding ferrous sulfate, age and alcohol consumption remained associated with supplementation. Pregnant people aged 30 years or older supplemented 6% more compared to those aged 18 to 19 years. On the other hand, those who consumed alcoholic beverages during pregnancy had 4% higher ferrous sulfate supplementation compared to those who did not consume alcohol. 

**Table 3:** Prevalence ratios (PR) from crude and adjusted analyses with 95% confidence intervals (95%CI) for folic acid and ferrous sulfate supplementation among pregnant women assisted in Primary Health Care in Criciúma, 2022 (n=428)

Variables	Folic acid		Ferrous sulfate	
	Crude	Adjusted	Crude	Adjusted
	PR (95%CI)	PR (95%CI)	PR (95%CI)	PR (95%CI)
**Age** (years)	0.206	0.159	0.011	0.006
18-19	1	1	1	1
20-29	0.96 (0.90; 1.01)	0.96 (0.91; 1.02)	1.0 (0.94; 1.08)	1.0 (0.94; 1.08)
≥30	0.94 (0.88; 1.01)	0.94 (0.86; 1.01)	1.05 (0.98; 1.12)	1.06 (0.99; 1.13)
**Race/skin color**	0.343	0.402	0.440	0.708
White	1	1	1	1
Black	0.94 (0.85; 1.04)	0.95 (0.85; 1.06)	0.98 (0.92; 1.04)	0.98 (0.92; 1.06)
Brown	0.98 (0.91; 1.04)	0.98 (0.91; 1.05)	0.99 (0.94; 1.03)	0.99 (0.95; 1.04)
**Marital status**	0.452	0.381	0.642	0.288
Single	1	1	1	1
Married/Stable Union	1.0 (0.94; 1.05)	1.0 (0.94; 1.05)	1.0 (0.97; 1.03)	0.98 (0.95; 1.02)
Separated/Divorced	0.88 (0.68; 1.14)	0.87 (0.64; 1.19)	0.94 (0.79; 1.11)	0.91 (0.75; 1.10)
Schooling	0.227	0.543	0.159	0.253
0-4	1	1	1	1
5-8	1.12 (0.78; 1.60)	1.11 (0.76; 1.61)	1.16 (0.80; 1.66)	1.15 (0.81; 1.63)
9-11	1.11 (0.78; 1.60)	1.08 (0.75; 1.57)	1.17 (0.81; 1.67)	1.17 (0.83; 1.66)
≥12	1.16 (0.80; 1.66)	1.12 (0.77; 1.62)	1.18 (0.82; 1.70)	1.17 (0.83; 1.66)
**Monthly income** (BRL)	0.120	0.162	0.115	0.237
<500.00	1	1	1	1
500.00-1,000.00	1.00 (0.90; 1.10)	1.00 (0.91; 1.09)	1.00 (0.94; 1.07)	1.00 (0.94; 1.06)
1,001.00-2,000.00	1.02 (0.94; 1.10)	1.02 (0.95; 1.09)	1.03 (0.98; 1.09)	1.03 (0.99; 1.08)
2,001.00-4,000.00	1.05 (0.97; 1.13)	1.05 (0.96; 1.14)	1.03 (0.97; 1.09)	1.02 (0.96; 1.08)
>4,000.00	1.09 (1.02; 1.16)	1.08 (1.00; 1.17)	1.05 (1.00; 1.10)	1.03 (0.98; 1.07)
**Number of visits**	0.020	0.022	0.132	0.183
<6 visits	1	1	1	1
Six or more visits	1.10 (1.01; 1.20)	1.10 (1.01; 1.20)	1.04 (0.99; 1.10)	1.04 (0.98; 1.10)
**Planned pregnancy**	0.384	0.484	0.956	0.709
No	1	1	1	1
Yes	1.02 (0.96; 1.07)	1.02 (0.96; 1.07)	1.0 (0.95; 1.06)	0.99 (0.96; 1.02)
**Smoking during pregnancy**	0.134	0.158	0.261	0.238
No	1	1	1	1
Yes	0.83 (0.63; 1.05)	0.85 (0.67; 1.07)	0.91 (0.78; 1.06)	0.91 (0.78; 1.06)
**Alcohol consumption**	0.852	0.944	<0.001	0.015
No	1	1	1	1
Yes	0,98 (0,88; 1,08)	1,01 (0,88; 1,16)	1,03 (1,01; 1,04)	1,04 (1,00; 1,08)

## Discussion 

The findings of this study indicate broad adherence to ferrous sulfate and folic acid supplementation among pregnant people attending Primary Health Care in the studied municipality (Criciúma). The higher prevalence of iron supplementation among older women and those who consumed alcohol during pregnancy suggests the influence of individual and behavioral characteristics on adherence to nutritional recommendations. In addition, the association between a greater number of prenatal visits and the use of folic acid reinforces the role of systematic follow-up in access to and adherence to interventions aimed at maternal-fetal health. These findings suggest the effectiveness of the strategies of the National Iron Supplementation Program (PNSF) in promoting supplementation.

This study has some limitations that should be considered. First, the cross-sectional design makes it impossible to infer causality between the associated factors and supplementation. In addition, the absence of information on the amount and regularity of the use of supplements in the prenatal booklet restricts the evaluation of full compliance with the recommendations of the National Iron Supplementation Program (PNSF). Despite these limitations, the study has relevant aspects, such as the representative sample size of pregnant people treated in Primary Health Care in Criciúma and the use of primary data taken directly from pregnant people’s prenatal booklets.

The high use of ferrous sulfate supplementation found in this study is higher than that reported in previous studies conducted in Brazil. A cross-sectional study conducted in Rio Grande, Rio Grande do Sul, with 2,557 women who had children in 2007, analyzed the sociodemographic characteristics and the assistance received during pregnancy and childbirth, indicating a prevalence of 59.0% of ferrous sulfate supplementation (19). Another study, also cross-sectional, with 70 pregnant women from Colombo, Paraná, which evaluated the association between the use of ferrous sulfate and folic acid in the last 30 days of pregnancy, showed a progressive increase in ferrous sulfate supplementation over the trimesters, reaching 90.9% in the third. (20). In the present study, we consider that the high rate of iron use may be explained by the data collection period, which was conducted in the third trimester of pregnancy, considering that pregnant people may have used the supplement at any point since conception. However, this data is also a possible indication of the effectiveness of the Primary Health Care policy in Criciúma, Santa Catarina.

The folic acid supplementation found in the present study showed a higher prevalence than that reported in previous studies (14,15). The study conducted in Rio Grande, Rio Grande do Sul, with 2,685 pregnant people, showed a prevalence of 54.2% of folic acid supplementation (15). The cohort study in Pelotas, Rio Grande do Sul, with 1,450 pregnant people, when evaluating folic acid supplementation, found a prevalence of 31.8% during pregnancy and 4.3% in the periconceptional period (21). 

The discrepancy between the findings of the present study and those of previous studies may be explained by differences in methodology, data collection period, and characteristics of the populations studied. The aforementioned Pelotas study, for example, included pregnant people from the private sector and those under 18 years old, which may have influenced the supplementation prevalence. 

The association between the mother’s age and ferrous sulfate supplementation, with higher use among pregnant people aged 30 years or older (PR 1.06; 95%CI 0.99; 1.13), corroborates findings from a birth cohort study conducted in Pelotas, Rio Grande do Sul, in 2015 (14). This finding can be explained by the fact that older women tend to postpone pregnancy and, therefore, may be more informed and aware of the importance of supplementation (22). Other studies, such as a cohort conducted in Maranhão (16), found higher ferrous sulfate use among younger pregnant people, possibly due to increased attention and greater recommendation by health personnel. The retrospective case-control study conducted in Pelotas, Rio Grande do Sul, showed that inadequate prenatal care was associated with multiparous pregnant people who had fewer visits than recommended, showing twice the risk of not completing the necessary prenatal visits compared to primiparous pregnant people. This stems from multiparous pregnant people relating previous experiences to those in the current pregnancy, creating greater confidence regarding pregnancy-related behaviors (23).

Alcohol consumption was also associated with higher ferrous sulfate supplementation, which may be explained by health personnel providing greater care to pregnant people in social vulnerability and/or those more likely to engage in risk behaviors (24,25). As demonstrated by the study conducted in Rio Grande, which analyzed sociodemographic factors and the care received by pregnant people during pregnancy and childbirth, the lowest prevalences of ferrous sulfate supplementation were observed among mothers with lower risk of adverse outcomes, as well as among non-adolescent mothers, those of White “race/skin color,” and those with a higher number of children (19).

For folic acid supplementation, women with six or more visits had a higher likelihood of supplementing. The results demonstrated the importance of the patient’s proximity to Primary Health Care, enabling better adherence of pregnant people to prophylactic measures. A study conducted with 280 women in Diamantina, Minas Gerais, found that 73.9% attended seven or more prenatal visits (25). A cross-sectional study conducted in Rio Branco, Acre, showed that of 887 primiparous pregnant people, 99.2% had access to prenatal care, and of these, 50.4% attended between six and eight visits (26). Another cross-sectional study, conducted in Rio Grande, Rio Grande do Sul, with 2,685 postpartum women, showed that 54.2% supplemented with folic acid, and 85.8% attended six or more prenatal visits (15). It is important to emphasize that an insufficient number of prenatal visits or late initiation of care can lead to problems such as increased morbidity and mortality, delayed diagnosis, and inadequate treatment of issues that may arise during the pregnancy and postpartum period (26).

In conclusion, the results indicated a high prevalence of ferrous sulfate and folic acid supplementation among pregnant people who attended Primary Health Care, suggesting the effectiveness of the National Iron Supplementation Program (PNSF) and the importance of prenatal care in promoting maternal-fetal health. Identifying factors associated with supplementation, such as maternal age, alcohol consumption during pregnancy, and number of prenatal visits, can guide more targeted and effective intervention strategies by health management. Continuous monitoring and improvement of the supplementation program are essential to ensure that all pregnant people have adequate access to these essential nutrients, thereby promoting better maternal and fetal health.
