# Supplementary material for: Ferrous Sulfate and Folic Acid Supplementation in Pregnant People in Primary Health Care: a Cross-Sectional Study, Criciúma, 2022
Source: Epidemiol Serv Saude. 2025 Nov 28;35:e20240377. doi: 10.1590/S2237-96222026v35e20240377.en (PMC12662406; doi:10.1590/S2237-96222026v35e20240377.en)
Supplement: Translation

# Translation: Suplementação com sulfato ferroso e ácido fólico em gestantes na Atenção Primária à Saúde: estudo transversal, Criciúma, 2022

**DOI:** 10.1590/S2237-96222026v35e20240377.pt

**Authors:** Jhonatan Campos-Rosa, Ricardo Augusto Cardoso Puchivailo, Antônio Augusto Schäfer, Fernanda de Oliveira Meller, Vanessa Iribarrem Avena Miranda

**Affiliations:** 1 Universidade do Extremo Sul Catarinense, Curso de medicina, Criciúma, SC, Brasil; 2 Universidade do Extremo Sul Catarinense, Programa de Pós-graduação em Saúde Coletiva, Criciúma, SC, Brasil

**Keywords:** Gestação, Ácido Fólico, Atenção Primária à Saúde, Sulfato Ferroso, Estudos transversais

## Abstract

**Objetivo:**

Avaliar a prevalência e os fatores associados à suplementação de sulfato ferroso e de ácido fólico em gestantes.

**Métodos:**

Estudo transversal, representativo, com gestantes atendidas na Atenção Primária à Saúde de Criciúma, Santa Catarina, no ano de 2023. As suplementações de sulfato ferroso e de ácido fólico foram avaliadas a partir da carteira de pré-natal das gestantes. A prevalência de suplementação foi calculada com seus respectivos intervalos de confiança de 95% (IC95%). Foram realizadas análises de regressão de Poisson bruta e ajustada (modelo hierárquico), estimando-se razões de prevalência (RP) e seus respectivos IC95%, para verificar os fatores associados.

**Resultados:**

Foram entrevistadas 428 gestantes. Dessas, 97,4% fizeram uso de suplementação de sulfato ferroso durante a gestação, e 93,7% de ácido fólico. As análises indicaram que a utilização do sulfato ferroso foi maior nas gestantes de maior idade, com RP 1,06 (IC95% 0,99; 1,13), e que consumiram bebida alcoólica durante a gestação, com RP 1,04 (IC95% 1,00; 1,08). Quanto ao uso do ácido fólico, este foi superior entre as gestantes que realizaram pelo menos seis consultas de pré-natal, com RP 1,10 (IC95% 1,01; 1,20).

**Conclusão:**

Os resultados apontam alta suplementação de ferro e de ácido fólico entre as gestantes da Atenção Primária à Saúde. A identificação dos fatores associados pode orientar estratégias de intervenção mais direcionadas e eficazes por parte da gestão de saúde.

**Aspectos éticos**

A pesquisa respeitou os princípios éticos, obtendo os seguintes dados de aprovação:

Comitê de ética em pesquisa: Universidade do Extremo Sul Catarinense

Número do parecer: 5.053.755

Data de aprovação: 22/10/2021

Certificado de apresentação de apreciação ética: 52547521.0.0000.0119

Registro do consentimento livre e esclarecido: Obtido de todos os participantes antes da coleta.

## Introdução 

A gravidez é um período de intensas mudanças fisiológicas em que diversas necessidades nutricionais são aumentadas para garantir o desenvolvimento saudável do feto e manter a saúde da gestante (1,2). Entre os nutrientes essenciais, o ferro e o ácido fólico possuem papel fundamental (2). A carência de ferro pode levar à anemia ferropriva, condição multifatorial que se manifesta quando há redução na quantidade de hemoglobina ou de eritrócitos, resultando na diminuição da capacidade do sangue de transportar oxigênio pelo organismo (3). A carência de ácido fólico durante o período pré-natal, por sua vez, também pode prejudicar o binômio materno-fetal, pois desempenha papel primordial na formação do tubo neural do feto, que se desenvolve nas primeiras semanas da gravidez, vindo a se tornar o cérebro e a medula espinhal do bebê (4,5).

É ideal que a maioria dos nutrientes seja obtida através de dieta equilibrada. Porém, as necessidades de ferro e de ácido fólico durante a gestação podem ser difíceis de serem atendidas apenas pela alimentação. Por esse motivo e pela alta prevalência de anemia em gestantes, especialmente em países em desenvolvimento, diversas organizações governamentais recomendam a suplementação desses micronutrientes durante a gravidez como estratégia para melhorar a condição nutricional e prevenir possíveis deficiências (6,7). 

No Brasil, o Programa Nacional de Suplementação de Ferro, implementado em 2005, preconiza a ingestão diária de ácido fólico por pelo menos 30 dias antes da concepção e até a 12ª semana de gestação (8). Quanto ao ferro, a recomendação é a sua administração diária da confirmação da gravidez até o terceiro mês após o parto (9,10).

A Atenção Primária à Saúde exerce papel central no âmbito do Sistema Único de Saúde, sendo a principal forma de acesso aos serviços de assistência médica pela população (11,12). É responsabilidade da equipe da Atenção Primária à Saúde a tarefa de realizar a estratificação de risco durante as consultas de pré-natal, classificando as gestantes de acordo com as suas peculiaridades e prevenindo possíveis déficits carenciais (13). Porém, mesmo com um programa de abrangência universal, estudos indicam que a suplementação diária está abaixo do recomendado, sendo preconizado 40 mg de ferro e 0,4 mg de ácido fólico (2,8,9). Dados do estudo de coorte, que avaliou 4.200 gestantes, mostraram que o uso de ácido fólico é mais frequente entre mulheres brancas, com maior renda e maior escolaridade. Em contrapartida, o uso profilático de ferro foi mais utilizado por mães não brancas, mais pobres e com menor escolaridade, sugerindo associação oposta para esse suplemento (14).

Existem vários estudos relatando o uso de suplementos na gestação. No entanto, poucos têm focado na Atenção Primária à Saúde (14-16). Embora haja o programa universal no Brasil para utilização de ácido fólico e de sulfato ferroso na gestação, a literatura aponta que o uso desses compostos pode estar abaixo do esperado (17). Como estratégia de avaliação do desempenho do programa, o presente estudo tem por objetivo investigar a prevalência e os fatores associados à suplementação de sulfato ferroso e de ácido fólico em gestantes atendidas na Atenção Primária à Saúde.

## Métodos 

### Delineamento do estudo

Trata-se de um estudo transversal, proveniente da pesquisa intitulada “Saúde mental e condições de vida das gestantes atendidas na Atenção Primária à Saúde”, no período de abril a dezembro de 2022. 

### Contexto 

Criciúma está localizado a cerca de 200 km ao sul de Florianópolis, capital de Santa Catarina, e possui em torno de 234,865 km^2^ de área territorial. Seu Índice de Desenvolvimento Humano é de 0,788. O município apresenta uma população de 215.186 habitantes, com rendimento mensal médio de 2,6 salários mínimos por pessoa e um produto interno bruto per capita de R$ 33.811,65 (18).

### Tamanho do estudo

O cálculo de tamanho de amostra foi realizado no programa Open Source Epidemiologic Statistics for Public Health (OpenEpi) com os seguintes parâmetros: nível de confiança de 95%, poder estatístico de 80% e prevalência do desfecho de 50%. Adicionou-se 10% para perdas e recusas e 15% para controle de fatores de confusão, totalizando um tamanho amostral estimado de 384 gestantes.

### Participantes 

A população alvo foi composta por gestantes com 18 anos ou mais, dentro do terceiro trimestre de gestação e que estivessem fazendo seu pré-natal em uma das 48 Unidades Básicas de Saúde de Criciúma. 

Como critérios de exclusão, foram considerados: gestantes menores de 18 anos; gestantes com déficit cognitivo ou com incapacidade de comunicação que as impedisse de responder ao questionário; gestantes de outra nacionalidade que não compreendessem a linguagem portuguesa; gestantes com gravidez de alto risco e/ou casos de aborto.

### Variáveis 

Os desfechos “suplementação de ferro” e “suplementação de ácido fólico” foram coletados do campo destinado a esse fim nas carteiras de pré-natal das gestantes, a qual era preenchida pelo profissional de saúde na consulta de rotina. As variáveis socioeconômicas utilizadas foram: idade materna (18-19 anos, 20-29 anos e ≥30 anos); “raça/cor da pele” materna das gestantes cadastradas no estudo (branca, preta/parda); estado civil (solteira, casada/união estável, separada); escolaridade materna (0-4 anos, 5-8 anos, 9-11 anos e ≥12 anos); e renda mensal (<500,00 reais, 500,00-1.000,00 reais, 1.001,00-2.000,00 reais, 2.001,00-4.000,00 reais e >4000,00 reais). Além disso, foram avaliados o planejamento da gravidez (não, sim) e o número de consultas realizadas durante o pré-natal (<6 consultas e ≥6 consultas).

### Fontes de dados e mensuração

A coleta de dados foi realizada por Agentes Comunitários de Saúde e por alunos da Residência Multiprofissional da Universidade do Extremo Sul Catarinense, os quais foram previamente treinados e orientados quanto à conduta no levantamento e no registro de dados. Os questionários foram aplicados utilizando-se o aplicativo Research Electronic Data Capture (RedCap) através de smartphones.

A identificação das gestantes no terceiro trimestre foi realizada através do sistema de saúde informatizado utilizado pelo município, chamado CELK saúde. Nele, eram gerados mensalmente os relatórios com data provável do parto para os próximos três meses, correspondentes ao terceiro trimestre de gestação. Todas as gestantes foram convidadas a participar do estudo. As entrevistas eram realizadas preferencialmente nas Unidades Básicas de Saúde, de acordo com a data da consulta pré-natal e, quando necessário, também eram realizadas na residência da gestante. 

### Controle de viés 

Para minimizar possíveis vieses, foram adotadas diversas estratégias metodológicas. A seleção das participantes foi realizada de forma sistemática, com base em relatórios mensais extraídos do sistema municipal CELK Saúde, garantindo a inclusão de gestantes no terceiro trimestre. A coleta foi conduzida por profissionais previamente treinados e padronizados, utilizando o aplicativo RedCap, o que reduziu erros de transcrição. As informações sobre suplementação foram extraídas das carteiras de pré-natal, uniformizando a fonte de dados. Contudo, reconhece-se como limitação o fato de esses registros indicarem apenas a prescrição, sem refletir a adesão real ou a regularidade do uso. 

### Métodos estatísticos

As análises foram realizadas no software Statistical Software for Data Science (STATA) versão 17.0, utilizando-se o módulo survey para amostras complexas que incorporam os pesos de pós-estratificação. Inicialmente, realizou-se a descrição da amostra em relação às variáveis independentes e calculou-se a prevalência dos desfechos de suplementação de ferro e de ácido fólico com os respectivos intervalos de confiança de 95% (IC95%).

Para verificar as associações entre as variáveis desfecho e explicativas, utilizou-se como medida de associação a razão de prevalência (RP), obtida por modelos de regressão de Poisson com variância robusta, seguindo-se o modelo hierárquico. O nível 1 incluiu as variáveis demográficas e socioeconômicas; o nível 2 as variáveis de hábitos de vida e planejamento da gravidez; e o nível 3 o número de consultas. Variáveis com p-valor<0,2 foram mantidas no modelo para controle de fatores de confusão. Considerou-se o nível de significância de 0,05.

## Resultados 

Foram avaliadas 428 gestantes (taxa de resposta de 85,6%). De acordo com a Tabela 1, a maioria pertencia ao grupo etário de 20 a 29 anos (53,3%), apresentava “raça/cor da pele” branca (68,1%) e estava casada ou em união estável (68,2%). Aproximadamente metade delas tinha de nove a 11 anos de estudo (50,5%). Cerca da metade tinha renda de R$ 1.001,00 a R$ 2.000,00 (45,0%). Quanto ao cuidado pré-natal, 79,7% realizaram seis ou mais consultas. A maioria das gestações não foi planejada (62,8%). Quanto aos hábitos de vida, a maioria das gestantes não era fumante (95,6%) e não fazia uso de bebidas alcoólicas (97,0%). 

**Tabela 1:** Distribuição da amostra com intervalo de confiança de 95% (IC95%) de acordo com as características das gestantes atendidas na Atenção Primária à Saúde. Criciúma, 2022 (n=428)

Variáveis	n (%)		(IC95%)
**Idade** (anos)			
18-19	42 (9,8)	(7,3; 13,0)	
20-29	228 (53,3)	(48,5; 58,0)	
≥30	158 (36,9)	(32,6; 41,6)	
**Raça/cor da pele**			
Branca	284 (68,1)	(63,5; 72,4)	
Preta	48 (11,5)	(8,8; 15,0)	
Parda	85(20,4)	(16,8; 24,5)	
Estado civil			
Solteira	124 (29,0)	(24,9; 33,5)	
Casada/união estável	292 (68,2)	(63,6; 72,5)	
Separada/divorciada	12 (2,8)	(1,6; 4,9)	
**Escolaridade (anos)**			
0-4	6 (1,4)	(0,6; 3,1)	
9-11	216 (50,5)	(45,7; 55,2)	
≥12	88 (20,5)	(17,0; 24,7)	
**Renda mensal (reais)**			
<500,00	86 (20,8)	(17,2; 25,0)	
500,00-1.000,00	70 (17,0)	(13,6; 20,1)	
1.001,00-2.000,00	186 (45,0)	(40,3; 49,9)	
2.001,00-4.000,00	57 (13,8)	(10,8; 17,5)	
>4.000,00	14 (3,4)	(2,0; 5,6)	
**Número de consultas de pré-natal**			
<6 consultas	87 (20,3)	(16,8; 24,4)	
Seis ou mais consultas	341 (79,7)	(75,6; 83,2)	
**Gravidez planejada**			
Não	269 (62,8)	(58,2; 67,3)	
Sim	159 (37,2)	(32,7; 41,8)	
Paridade			
1	129 (48,9)	(42,8; 54,9)	
2	79 (29,9)	(24,6; 35,7)	
3	35 (13,2)	(9,6; 17,9)	
4 ou mais	21 (8,0)	(5,2; 11,9)	
**Fumo durante a gestação**			
Não	409 (95,6)	(93,2; 97,2)	
**Consumo de bebida alcoólica**			
Não	414 (97,0)	(94,8; 98,2)	
Sim	13 (3,0)	(1,8; 5,2)	

Durante a gestação, 97,4% das mulheres realizaram a suplementação de sulfato ferroso, enquanto 93,7% suplementaram o ácido fólico. Quando as suplementações foram avaliadas de acordo com as características das gestantes, foi possível perceber relação direta entre idade e uso de suplemento de ferro. Conforme a idade avança, houve aumento na prevalência, chegando a 100% em gestantes de 30 anos ou mais (p-valor 0,035). Além disso, as gestantes que passaram por seis ou mais consultas de pré-natal apresentaram maior uso de sulfato ferroso, alcançando prevalência de 98,2%. Já aquelas que realizaram menos de seis consultas, tiveram menor prevalência de suplementação de ferro (94,2%) (p-valor 0,036). 

A mesma tendência foi observada em relação à suplementação de ácido fólico. As gestantes que realizaram seis ou mais consultas de pré-natal apresentaram maior frequência de uso, atingindo 95,6%, enquanto aquelas que realizaram menos de seis consultas registraram 86,2% (p-valor 0,001). Em relação ao tabagismo durante a gestação, os resultados apontaram que as gestantes fumantes suplementaram menos com ferro (89,5%), em contraste com as não-fumantes (97,8%) (p-valor 0,025). No que se diz respeito ao ácido fólico, a mesma característica foi observada, com as gestantes fumantes apresentando frequência de uso mais baixa, de 78,9%, enquanto as não-fumantes apresentaram frequência de 94,4% (p-valor 0,007), conforme Tabela 2.

**Tabela 2:** Prevalência de suplementação de ácido fólico e de ferro de acordo com as características das gestantes atendidas na Atenção Primária à Saúde. Criciúma, 2022 (n=428)

Variáveis	Suplementação de ácido fólico		Suplementação de ferro	
	n (%)	p-valor	n (%)	p-valor
**Idade (anos)**		0,461		0,035
18-19	41 (97,6)		40 (95,2)	
20-29	214 (93,9)		219 (96,0)	
≥30	146 (92,4)		158 (100)	
**Raça/cor da pele**		0,300		0,605
Branca	270 (95,1)		278 (98,9)	
Preta	43 (89,6)		46 (95,8)	
Parda	79 (93,0)		82 (96,5)	
**Estado civil**		0,320		0,441
Solteira	117 (94,3)		121 (97,6)	
Casada/união estável	274 (93,8)		285 (97,6)	
Separada/divorciada	10 (83,3)		11 (91,7)	
**Escolaridade (anos)**		0,469		0,119
0-4	5 (83,3)		5 (83,3)	
5-8	110 (93,2)		114 (96,6)	
9-11	201 (93,1)		211 (97,7)	
≥12	85 (96,6)		87 (98,9)	
**Renda mensal (reais)**		0,614		0,497
<500,00	79 (91,9)		82 (95,35)	
500,00-1.000,00	64 (91,4)		67 (95,7)	
1.001,00-2.000,00	174 (93,6)		183 (98,4)	
2.001,00-4.000,00	55 (96,5)		56 (98,2)	
>4.000,00	14 (100)		14 (100)	
**Número de consultas**		0,001		0,036
<6 consultas	75 (86,2)		82 (94,2)	
Seis ou mais consultas	326 (95,6)		335 (98,2)	
**Gravidez planejada**		0,403		0,956
Não	250 (92,9)		262 (97,4)	
Sim	151 (95,0)		155 (97,5)	
Paridade		0,063		0,665
1	120 (49,6)		124 (48,6)	
2	74 (30,6)		77 (30,2)	
3	32 (13,2)		33 (12,9)	
4 ou mais	16 (6,6)		21 (8,2)	
**Fumo durante a gestação**		0,007		0,025
Não	386 (94,4)		400 (97,8)	
Sim	15 (78,9)		17 (89,5)	
**Consumo de bebida alcoólica**		0,837		0,552
Não	388 (93,7)		403 (97,3)	
Sim	12 (92,3)		13 (100)	

A Tabela 3 descreve os fatores associados à suplementação durante a gestação. Quanto ao ácido fólico, a análise bruta e ajustada apontou associação com número adequado de consultas de pré-natal. Gestantes com seis ou mais consultas apresentaram suplementação de ácido fólico 10% maior quando comparadas àquelas com menos de seis consultas. Em relação ao sulfato ferroso, permaneceram associados à suplementação a idade e o consumo de bebida alcoólica. Gestantes com idade maior ou igual a 30 anos suplementam 6% a mais quando comparadas àquelas de 18 a 19 anos de idade. Já aquelas que consumiram bebida alcoólica durante a gestação tiveram 4% mais suplementação de sulfato ferroso quando comparadas àquelas que não consumiram. 

**Tabela 3:** Razões de prevalência (RP) das análises brutas e ajustadas, com intervalo de confiança de 95% (IC95%) para suplementação de ácido fólico e de sulfato ferroso das gestantes atendidas na Atenção Primária à Saúde. Criciúma, 2022 (n=428)

Variáveis	Ácido fólico		Sulfato ferroso	
	OR Bruta	OR Ajustada	OR Bruta	OR Ajustada
	RP (IC95%)	RP (IC95%)	RP (IC95%)	RP (IC95%)
**Idade (anos)**	0,206	0,159	0,011	0,006
18-19	1	1	1	1
20-29	0,96 (0,90; 1,01)	0,96 (0,91; 1,02)	1,0 (0,94; 1,08)	1,0 (0,94; 1,08)
≥30	0,94 (0,88; 1,01)	0,94 (0,86; 1,01)	1,05 (0,98; 1,12)	1,06 (0,99; 1,13)
**Raça/cor da pele**	0,343	0,402	0,440	0,708
Branca	1	1	1	1
Preta	0,94 (0,85; 1,04)	0,95 (0,85; 1,06)	0,98 (0,92; 1,04)	0,98 (0,92; 1,06)
Parda	0,98 (0,91; 1,04)	0,98 (0,91; 1,05)	0,99 (0,94; 1,03)	0,99 (0,95; 1,04)
**Estado civil**	0,452	0,381	0,642	0,288
Solteira	1	1	1	1
Casada/união estável	1,0 (0,94; 1,05)	1,0 (0,94; 1,05)	1,0 (0,97; 1,03)	0,98 (0,95; 1,02)
Separada/divorciada	0,88 (0,68; 1,14)	0,87 (0,64; 1,19)	0,94 (0,79; 1,11)	0,91 (0,75; 1,10)
Escolaridade	0,227	0,543	0,159	0,253
0-4	1	1	1	1
5-8	1,12 (0,78; 1,60)	1,11 (0,76; 1,61)	1,16 (0,80; 1,66)	1,15 (0,81; 1,63)
9-11	1,11 (0,78; 1,60)	1,08 (0,75; 1,57)	1,17 (0,81; 1,67)	1,17 (0,83; 1,66)
≥12	1,16 (0,80; 1,66)	1,12 (0,77; 1,62)	1,18 (0,82; 1,70)	1,17 (0,83; 1,66)
**Renda mensal (reais)**	0,120	0,162	0,115	0,237
<500,00	1	1	1	1
500,00-1.000,00	1,00 (0,90; 1,10)	1,00 (0,91; 1,09)	1,00 (0,94; 1,07)	1,00 (0,94; 1,06)
1.001,00-2.000,00	1,02 (0,94; 1,10)	1,02 (0,95; 1,09)	1,03 (0,98; 1,09)	1,03 (0,99; 1,08)
2.001,00-4.000,00	1,05 (0,97; 1,13)	1,05 (0,96; 1,14)	1,03 (0,97; 1,09)	1,02 (0,96; 1,08)
>4.000,00	1,09 (1,02; 1,16)	1,08 (1,00; 1,17)	1,05 (1,00; 1,10)	1,03 (0,98; 1,07)
**Número de consultas**	0,020	0,022	0,132	0,183
<6 consultas	1	1	1	1
Seis ou mais consultas	1,10 (1,01;1,20)	1,10 (1,01; 1,20)	1,04 (0,99; 1,10)	1,04 (0,98; 1,10)
**Gravidez planejada**	0,384	0,484	0,956	0,709
Não	1	1	1	1
Sim	1,02 (0,96; 1,07)	1,02 (0,96; 1,07)	1,0 (0,95; 1,06)	0,99 (0,96; 1,02)
**Fumo durante a gestação**	0,134	0,158	0,261	0,238
Não	1	1	1	1
Sim	0,83 (0,63; 1,05)	0,85 (0,67; 1,07)	0,91 (0,78; 1,06)	0,91 (0,78; 1,06)
**Consumo de bebida alcoólica**	0,852	0,944	<0,001	0,015
Não	1	1	1	1
Sim	0,98 (0,88; 1,08)	1,01 (0,88; 1,16)	1,03 (1,01; 1,04)	1,04 (1,00; 1,08)

## Discussão 

Os achados deste estudo indicam ampla adesão à suplementação de sulfato ferroso e de ácido fólico entre as gestantes atendidas na Atenção Primária à Saúde do município estudado (Criciúma). A maior prevalência de suplementação de ferro entre as mulheres mais velhas e aquelas que consumiram bebidas alcoólicas durante a gestação sugere a influência de características individuais e comportamentais na adesão às recomendações nutricionais. Além disso, a associação entre um maior número de consultas pré-natais e o uso de ácido fólico reforça o papel do acompanhamento sistemático no acesso e na adesão às intervenções voltadas à saúde materno-fetal. Esses achados sugerem efetividade das estratégias do Programa Nacional de Suplementação de Ferro na promoção da suplementação.

Este estudo apresenta algumas limitações que devem ser consideradas. Primeiramente, o delineamento transversal impossibilita a inferência de causalidade entre os fatores associados e a suplementação. Além disso, a ausência de informações sobre a quantidade e a regularidade do uso dos suplementos na carteira de pré-natal das gestantes restringe a avaliação do cumprimento integral das recomendações do Programa Nacional de Suplementação de Ferro. Apesar dessas limitações, o estudo possui aspectos relevantes, como o tamanho amostral representativo das gestantes atendidas na Atenção Primária à Saúde de Criciúma e a utilização de dados primários retirados diretamente das carteiras de pré-natal.

A alta utilização de suplementação de sulfato ferroso encontrada neste estudo é superior à de estudos anteriores realizados no Brasil. Um estudo transversal, realizado em Rio Grande, no Rio Grande do Sul, com 2.557 mulheres que tiveram filhos em 2007, analisou as características sociodemográficas e a assistência recebida durante a gestação e o parto, apontando prevalência de 59,0% de suplementação de sulfato ferroso (19). Outro estudo, também transversal, com 70 gestantes de Colombo, Paraná, que avaliou a associação entre o uso de sulfato ferroso e de ácido fólico nos últimos 30 dias da gestação, apresentou aumento progressivo na suplementação de sulfato ferroso ao longo dos trimestres, atingindo 90,9% no terceiro. (20). No presente estudo consideramos que o elevado índice do uso de ferro pode ser justificado pelo período da coleta dos dados, realizado no terceiro trimestre gestacional, levando em conta que a gestante fez uso do suplemento em qualquer momento desde a concepção. Entretanto, esse dado também é um possível indicativo da efetividade da Política de Atenção Primária à Saúde de Criciúma, Santa Catarina.

A suplementação de ácido fólico encontrada pelo presente estudo apresentou prevalência superior do que a achada em estudos prévios (14,15). O estudo realizado em Rio Grande, Rio Grande do Sul, com 2.685 gestantes, apresentou prevalência de 54,2% de suplementação de ácido fólico (15). Já o estudo de coorte em Pelotas, Rio Grande do Sul, com 1.450 gestantes, ao avaliar a suplementação de ácido fólico, encontrou prevalência de 31,8% durante a gestação e de 4,3% no período periconcepcional (21). 

A discrepância entre os achados do presente estudo e os de estudos anteriores pode ser explicada por diferenças na metodologia, no período de coleta de dados e nas características das populações estudadas. O citado estudo de Pelotas, por exemplo, incluiu gestantes da rede privada e menores de 18 anos, o que pode ter influenciado a prevalência de suplementação. 

A associação entre idade materna e suplementação de sulfato ferroso, com um maior uso entre gestantes com 30 anos ou mais RP 1,06 (IC95% 0,99; 1,^13^), corrobora achados de um estudo de coorte de nascimentos realizado em Pelotas, Rio Grande do Sul, em 2015 (14). Esse achado pode ser explicado pelo fato de que mulheres com idade mais avançada tendem a adiar a gestação e, portanto, podem estar mais informadas e conscientes sobre a importância da suplementação (22). Outros estudos, como um de coorte realizado no Maranhão (16), encontraram maior uso de sulfato ferroso entre gestantes mais jovens, possivelmente devido a uma maior atenção e maior recomendação por parte dos profissionais de saúde. O estudo caso-controle retrospectivo realizado em Pelotas, Rio Grande do Sul, mostrou que o acompanhamento pré-natal inadequado, com número de consultas inferior ao recomendado, esteve relacionado com gestantes multíparas, tendo estas demonstrado duas vezes maior risco de não concluir as consultas de pré-natal necessárias quando comparadas às primigestas. Isso decorre de gestantes multíparas associarem as experiências anteriores com as que estão vivenciando na gestação atual, criando maior segurança quanto às atitudes voltadas a ela (23).

O consumo de bebida alcoólica também foi associado a uma maior suplementação de sulfato ferroso, o que pode ser explicado pelo fato de o profissional de saúde promover maior cuidado a gestantes em vulnerabilidade social e/ou àquelas com maiores chances de apresentarem comportamentos de risco (24,25). Como demonstrou o estudo realizado em Rio Grande, que analisou fatores sociodemográficos e a assistência que as gestantes receberam durante a gestação e o parto, as menores prevalências observadas em relação à suplementação com sulfato ferroso foram em mães que apresentaram menores riscos quanto à ocorrência de desfechos desfavoráveis, além de mães não adolescentes, de “raça/cor da pele” branca e com maior número de filhos (19).

Na suplementação de ácido fólico, as mulheres com seis ou mais consultas apresentaram maior probabilidade de suplementar. Os resultados demonstraram a importância da proximidade da paciente com a Atenção Primária à Saúde, proporcionando melhor adesão da gestante às medidas profiláticas. Estudo realizado com 280 mulheres de Diamantina, Minas Gerais, apontou que 73,9% frequentaram sete ou mais consultas de pré-natal (25). Já um estudo transversal realizado em Rio Branco, Acre, demonstrou que, de 887 gestantes primigestas, 99,2% tiveram acesso ao pré-natal e, destas, 50,4% conseguiram realizar entre seis e oito consultas (26). Outro estudo transversal, este realizado em Rio Grande, Rio Grande do Sul, com 2.685 puérperas, mostrou que 54,2% delas realizaram a suplementação de ácido fólico, tendo 85,8% realizado seis ou mais consultas de pré-natal (15). Ressalta-se que o número insuficiente de consultas no pré-natal ou o início tardio delas pode desencadear problemas, como o aumento da morbimortalidade, o atraso de diagnóstico e o tratamento inadequado de problemas que possam ocorrer no período gravídico-puerperal (26).

Em conclusão, os resultados indicaram alta prevalência de suplementação de sulfato ferroso e de ácido fólico entre gestantes atendidas na Atenção Primária à Saúde, sugerindo eficácia do Programa Nacional de Suplementação de Ferro e a importância do acompanhamento pré-natal na promoção da saúde materna e fetal. A determinação de fatores associados à suplementação, como idade materna, consumo de bebida alcoólica durante a gestação e número de consultas de pré-natal, pode orientar estratégias de intervenção mais bem direcionadas e eficazes por parte da gestão de saúde. A contínua monitorização e o aprimoramento do programa de suplementação são fundamentais para garantir que todas as gestantes tenham acesso adequado a esses nutrientes essenciais, promovendo, assim, uma saúde materna e fetal de melhor qualidade.
